# Circulating microRNAs in aging

**DOI:** 10.18632/oncotarget.3175

**Published:** 2015-01-13

**Authors:** Massimiliano Bonafè, Fabiola Olivieri

**Affiliations:** Department of Clinical and Molecular Sciences, DISCLIMO, Università Politecnica delle Marche, and Center of Clinical Pathology and Innovative Therapy, INRCA-IRCCS National Institute, Ancona, Italy; Department of Experimental, Diagnostic and Specialty Medicine, DIMES, University of Bologna, Bologna, Italy

Aging research is currently focusing on circulating functional markers that can help discriminate the mechanisms of physiological aging from those of age-related diseases (ARDs). The cellular model of choice for this type of studies, cell senescence, has been providing highly valuable information.

Senescent cells are characterized by permanent cell cycle arrest and acquisition of a senescence-associated secretory phenotype (SASP) [1]. This identifiable secretome can propagate senescence to surrounding cells and contribute to inflamm-aging, the systemic pro-inflammatory drift that accompanies aging and ARDs [2].

Micro (mi)RNAs are short, single-stranded RNAs that are increasingly recognized as epigenetic regulators of gene expression, modulating all cell functions including senescence [3]. These mediators of epigenetic information, which are actively secreted by living cells and circulate in all body fluids, are extensively being investigated as potential ARD biomarkers [4].

Our group recently reported a senescence-related increase of plasma miR-126-3p in healthy aged individuals and increased miR-126-3p synthesis and secretion in endothelial cells undergoing senescence [5]. Interestingly, plasma from type 2 diabetes (T2D) patients and endothelial cells exposed to hyperglycemic media shared reduced miR-126-3p concentrations. We also found that senescent endothelial cells exhibit significant downregulation of miR-126-3p target SPRED-1, an inhibitor of angiogenic signaling [5]. Notably, hyperglycemic conditions hamper miR-126-3p/SPRED-1 axis modulation [5].

Overall, our investigations have provided some interesting insights: i) they have unveiled an endothelial epigenetic remodeling program aimed at maintaining vascular homeostasis during aging that can be monitored by circulating miR-126-3p; ii) they suggest that miR-126-3p may be one of the mechanisms by which loss of replicative and survival capacity is at least partly offset by cells undergoing replicative senescence; iii) they indicate that the miR-126-3p-dependent mechanism is blunted in endothelial cells exposed to high glucose concentrations, a phenomenon that is reminiscent of the increased risk of micro and macrovascular complications experienced by T2D patients; and iv) they extend and confirm the notion that miR-126-3p-related endothelial dysfunction can be studied *in vitro* through the mechanisms of endothelial cell senescence.

MiR-126 has a role in the maintenance of endothelial integrity, enhancing endothelial function and promoting blood vessel formation [6]. Intriguingly, recent studies have identified it as a modulator of inflammation and innate immune response, targeting some components of the nuclear factor (NF)-kB pathway, the master regulator of the pro-inflammatory program [7]. Our data therefore also provide proof of principle that miRNAs can be active components of the senescent endothelial cell secretome and that senescence-associated miRNAs (SA-miRNAs) may modulate the rate of inflamm-aging at the cellular and systemic level.

In this framework, the compartmentalization of circulating miRNAs into different shuttles (proteins, lipoproteins, exosomes) is likely to constitute a biological communication code among tissues and organs that needs to be deciphered. Notably, miR-126 shuttled by exosomes is biologically active in target cells, strongly supporting the hypothesis that exosomal miRNAs have important roles in cellular cross-talk, potentially affecting ARDs progression.

In the near future the identification of SASP-related miRNAs (and of other non-coding RNAs) and their specific shuttles, an aspect that has not yet been extensively investigated, is expected not only to help clarify the complex mechanisms of aging, but also to enable prediction or early diagnosis of the major ARDs and ultimately to inspire innovative strategies to delay their onset.
